# Genetic diagnosis of X-linked dominant hypophosphatemic rickets in a cohort study: Tubular reabsorption of phosphate and 1,25(OH)_2_D serum levels are associated with *PHEX *mutation type

**DOI:** 10.1186/1471-2350-12-116

**Published:** 2011-09-08

**Authors:** Marcos Morey, Lidia Castro-Feijóo, Jesús Barreiro, Paloma Cabanas, Manuel Pombo, Marta Gil, Ignacio Bernabeu, José M Díaz-Grande, Lourdes Rey-Cordo, Gema Ariceta, Itxaso Rica, José Nieto, Ramón Vilalta, Loreto Martorell, Jaime Vila-Cots, Fernando Aleixandre, Ana Fontalba, Leandro Soriano-Guillén, José M García-Sagredo, Sixto García-Miñaur, Berta Rodríguez, Saioa Juaristi, Carmen García-Pardos, Antonio Martínez-Peinado, José M Millán, Ana Medeira, Oana Moldovan, Angeles Fernandez, Lourdes Loidi

**Affiliations:** 1 Fundación Pública Galega de Medicina Xenómica, Santiago de Compostela, Spain; 2 Unidad de Endocrinología Pediátrica, Crecimiento y Adolescencia. Pediatría, Hospital Clínico Universitario y Universidad de Santiago de Compostela, Spain; 3 Pediatría. Hospital Clínico Universitario, Santiago de Compostela, Spain; 4 Endocrinología, Hospital Clínico Universitario, Santiago de Compostela, Spain; 5 Pediatría, Complejo Hospitalario de Pontevedra, Spain; 6 Pediatría, Complejo Hospitalario de Vigo, Spain; 7 Pediatría, Hospital de Cruces, Barakaldo, Spain; 8 Nefrología Pediátrica, Hospital Vall d'Hebrón, Barcelona, Spain; 9 Genética, Hospital San Joan de Deu, Espluges-Barcelona, Spain; 10 Nefrología, Hospital San Joan de Deu, Espluges-Barcelona, Spain; 11 Pediatría, Hospital Virgen de la Salud, Elda, Spain; 12 Genética, Hospital Universitario Marqués de Valdecilla, Santander, Spain; 13 Pediatría, Fundación Jiménez Díaz, Madrid, Spain; 14 Genética, Hospital Ramón y Cajal, Madrid, Spain; 15 Genética, Hospital Universitario La Paz, Madrid, Spain; 16 Genética, Complejo Hospitalario A Coruña, Spain; 17 Genética, Hospital Donostia, San Sebastián, Spain; 18 Pediatría, Hospital Donostia, San Sebastián, Spain; 19 Genética, Hospital Reina Sofía, Córdoba, Spain; 20 Genética, Hospital La Fé and CIBERER, Valencia, Spain; 21 Genética, Hospital Santa María, Lisboa, Portugal; 22 Pediatría, Complejo Hospitalario de Toledo. Spain

## Abstract

**Background:**

Genetic Hypophosphatemic Rickets (HR) is a group of diseases characterized by renal phosphate wasting with inappropriately low or normal 1,25-dihydroxyvitamin D_3 _(1,25(OH)_2_D) serum levels. The most common form of HR is X-linked dominant HR (XLHR) which is caused by inactivating mutations in the *PHEX *gene. The purpose of this study was to perform genetic diagnosis in a cohort of patients with clinical diagnosis of HR, to perform genotype-phenotype correlations of those patients and to compare our data with other HR cohort studies.

**Methods:**

Forty three affected individuals from 36 non related families were analyzed. For the genetic analysis, the *PHEX *gene was sequenced in all of the patients and in 13 cases the study was complemented by mRNA sequencing and Multiple Ligation Probe Assay. For the genotype-phenotype correlation study, the clinical and biochemical phenotype of the patients was compared with the type of mutation, which was grouped into clearly deleterious or likely causative, using the Mann-Whitney and Fisher's exact test.

**Results:**

Mutations in the *PHEX *gene were identified in all the patients thus confirming an XLHR. Thirty four different mutations were found distributed throughout the gene with higher density at the 3' end. The majority of the mutations were novel (69.4%), most of them resulted in a truncated PHEX protein (83.3%) and were family specific (88.9%). Tubular reabsorption of phosphate (TRP) and 1,25(OH)_2_D serum levels were significantly lower in patients carrying clearly deleterious mutations than in patients carrying likely causative ones (61.39 ± 19.76 vs. 80.14 ± 8.80%, p = 0.028 and 40.93 ± 30.73 vs. 78.46 ± 36.27 pg/ml, p = 0.013).

**Conclusions:**

*PHEX *gene mutations were found in all the HR cases analyzed, which was in contrast with other cohort studies. Patients with clearly deleterious *PHEX *mutations had lower TRP and 1,25(OH)_2_D levels suggesting that the *PHEX *type of mutation might predict the XLHR phenotype severity.

## Background

Genetic Hypophosphatemic Rickets (HR) is a group of diseases characterized by renal phosphate wasting, with inappropriately low or normal serum 1,25-dihydroxyvitamin D_3 _(1,25(OH)_2_D) levels, causing growth retardation, rickets and osteomalacia. The most common form is X-linked dominant hypophosphatemic rickets (XLHR, OMIM 307800) with an incidence of 1/20,000 [1]. XLHR is caused by inactivating mutations in the *PHEX *gene (Phosphate Regulating Gene with Homologies to Endopeptidases on the × chromosome) which is located in Xp22.1-22.2 [2]. Singular cases are autosomal forms with a much lesser incidence. These include autosomal dominant HR, caused by mutations in the fibroblast growth factor 23 gene (*FGF23*) (ADHR, OMIM 193100) [3] and autosomal recessive HR caused by mutations in dentin matrix protein 1 gene (*DMP1*) (ARHR1, OMIM 241520) [4] and mutations in ectonucleotide pyrophosphatase/phosphodiesterase-1 gene (*ENPP1*) (ARHR2, OMIM 613312) [5,6]. Excess action of fibroblast growth factor 23 (FGF23) underlies the pathogenesis of these hypophosphatemic diseases. FGF23 acts mainly as a phosphaturic factor [7] and also reduces the 1,25(OH)_2_D production [8]. Although initially FGF23 was suggested as a substrate for PHEX, further studies failed to confirm this [9,10]. On the other hand, PHEX binds to MEPE C-terminal ASARM peptide and neutralizes its biological activity as an inhibitor of mineralization [11,12].

The *PHEX *gene consists of 22 short exons with huge intronic regions. The coding region spans 2,250 bp and encodes for a 749-aminoacid transmembrane endopeptidase that belongs to the type II integral membrane zinc-dependent endopeptidase family [13].

Structurally, PHEX has a small aminoterminal intracellular tail, a short transmembrane domain and a large carboxyterminal extracellular domain with the catalytic and zinc-binding sites [14,15]. PHEX is mainly expressed in osteoblast, osteocytes and odontoblast [16,17].

More than 250 different *PHEX *mutations have been described to date, including nonsense (17-19%), missense (21-22%), deletions (24-30%), insertions (11-12%) and splice mutations (18-24%) unevenly distributed along the *PHEX *gene with three regions with high mutation density [18-20].

The main purpose of this study was to perform genetic diagnosis in a cohort of patients with clinical diagnosis of HR. The second objective was to asses any possible genotype-phenotype correlation. The third was to review and compare the prevalence and distribution of *PHEX *mutations in the present study with previously published HR cohort studies.

## Methods

### Patient's phenotype

A total of 46 patients clinically diagnosed with HR and one pre-symptomatic infant (11 males and 35 females) belonging to 36 unrelated families were studied. The parents of the children patients or the patient itself, who had given the informed consent to participate in the study, were referred to the laboratory for genetic analysis from different Spanish and Portuguese hospitals. Forty six non affected family members were also analyzed. The age at diagnosis of the 36 probands as well as the clinical and biochemical features are summarized in table 1. Samples were collected after 12 hours of fasting and the assessment of phosphate serum levels (P) (Normal values: adolescents and adults > 2.5, infants 4.8-7.4; toddlers 4.5-5.8; children 3.5-5.5 mg/dl [21]), tubular reabsorption of phosphate (TRP), 25 hydroxy vitamin D (25(OH)D), 1,25 dihydroxyvitamin D (1,25(OH)_2_D), parathyroid hormone (PTH) and alkaline phosphatase (AP) were performed in each referring hospital. All the measurements were at the time of diagnosis and prior to any treatment. The severity of skeletal deformities was qualitatively assessed by the physician into: mild: patients with null or subtle genu varum, moderate: genu varum and/or femur deformities not requiring correcting surgery and severe: genu varum and/or femur deformities requiring correcting surgery. The patient's height SDS [22], at the time of diagnosis and the presence of nephrocalcinosis and/or hyperparathyroidism before and after treatment were also recorded. It was impossible to get detailed phenotypic data from some patients in spite of a clinical diagnosis of HR for all of them.

**Table 1:** Phenotype characteristic of studied HR patients

Pr	G	AD	BPS	H	P (mg/dl)	TRP (%)	25(OH)D (ng/mL)	1,25(OH)2D (pg/ml)	PTH (pg/mL)	AP (UI/l)	N	HP
1	F	1 y 3 m	Mild		2.9		28	87		1531		
2	F	3 y	Sev	-2.71	2.3	60			48	558	N	Y
3	M	9 y	Sev	-3.38	2.3	56.4		13.2	21	766	N	N
4	M	1 y 4 m	Sev	-2.34	2.8	78.4	29.7	73	28	378	N	N
5	F	1 y 4 m			2.7	25.3	12				Y	
6	F	1 y 10 m	Mod	-4.06	2.1						N	N
7	M	2 y 3 m	Mod	-3.2	2.9	76	175.2		79	1786	Y	N
8	M	1 y 5 m	Mod								N	N
9	M	2 y 6 m	Mod	-2.01	2.4	80	79	38.2	18	584	Y	Y
10	F	1 y 6 m	Mod	-2.05	2.7	91			52	791	N	N
11	F	4 m	Mild	0.88	4	89	41	75	75	1755	N	N
12	F	1 y 6 m	Sev	-3.35	2.1	32	27.5	16	68	1646	N	N
13	F	7 y	Sev	-2.24	2.8	58.2		21.9	49	1699	N	Y
14	F	3 y										
15	F											
16	F	4 y 6 m	Mod	-1.97	2.5	52		28	29	991	N	Y
17	F	4 y 7 m	Sev	-4.86			16	28	25	755	N	Y
18	F	1 y 7 m	Mod	-3.11	3.1	82.3	43	60	60	934	N	N
19	F	14 y	Sev	-3.84		66	37.5		42	534	N	N
20	F	1 y 7 m	Mild	-2.53	2.2	71	30.1	129	66.7	597	N	Y
21	F	2 y 8 m	Sev	-2.97	2.5	78	28	53	66	1020	N	N
22	F	4 y	Sev	-1.65	2.1	83			23	448	Y	N
23	M	3 y 2 m	Mod	-5.47	2.7	23					Y	BT
24	M	1 y 2 m	Sev	-2.26	2.0	59		30.1	57	1010	Y	Y
25	F	2 y 6 m	Sev	-2	2.7	46	45.2	53.4	48	528	N	Y
26	F	1 y 4 m	Mild	0.62	2.5		73	76	60	1249	N	N
27	M	3 y 2 m	Mod	-3.23	2.6	77.6	30	41	43	1389	N	N
28	F	2 y 6 m	Mild	-2.56	2.8	33				220	N	N
29	F	1 y 6 m	Mod	-3.1	2.4	66	54.2	39.8	58	856	N	N
30	F	4 y 10 m	Mod	-1.84	2.1	85	18	147	78	1513	N	BT
31	F											
32	F	5 y	Sev	-3.31	3.1	59.2		32	52.3	1110	N	Y
33	M	5 m	Sev	0.64	3.7	62.3	6.6	13.8	22	1204	N	N
34	F	3 y	Mild	-4.37	2.2						N	N
35	F	3 y 8 m	Mod	-2.3	2.6	75	39	14	49	1109	N	N
36	F	4 y 5 m	Sev	-1.95	3.7	86.7	31	56.3	49	470	N	N

Pr proband, G gender, AD age at diagnosis, BPS bone phenotype severity (Mod moderate, Sev severe), H height SDS, P phosphate serum levels, TRP tubular reabsorption of phosphate, 1,25(OH)_2_D 1,25 dihydroxi vitamin D, 25(OH)D 25 hydroxy vitamin D, PTH parathyroid hormone at diagnosis, AP alkaline phosphatase, N nephrocalcinosis after treatment, HP hyperparathyroidism (Y postreatment, BT before treatment, N no)

All the procedures followed were in accordance with the local legislation and with the current Helsinki Declaration.

### Mutational analysis of the PHEX gene

DNA isolation from peripheral blood leukocytes was carried out in the Chemagic Magnetic Separation Module I (Chemagen, Baesweiler, Germany) following the manufacturer's instructions. RNA was obtained from peripheral blood using the RNeasy mini Kit (Qiagen, Valencia CA, USA). For the detection of punctual mutations, the 22 exons and intron exon boundaries that comprise the *PHEX *gene were independently amplified by PCR using primers already described [23]. The complete *PHEX *coding region was amplified by RT-PCR in three overlapping fragments with the following pairs of primers: 5' ATGGAAGCAGAAACAGGGAG 3' and 5' GCTGTATTGACCACGAAACG 3', 5' AGAAAGCCAAAATCCTTTAT 3' and 5' TTGGTGGATGCACTGTAGAA 3' and 5' GCCGACTACTTTGGCAACGT 3' and 5' CTACCAGAGTCGGCAGGAGT 3'. The PCR products were purified and both strands were subjected to cycle sequencing using the BigDye terminator kit and run in the 3730 × l DNA Analyzer (Applied Biosystems, Foster City, CA, USA). The obtained electropherograms were analyzed with the Staden Package V.1.6 (http://www.staden.sourceforge.net). For the detection of exons deletions or duplications Multiple Ligation Dependent Probe Amplification (MLPA) was used following the manufacturer's recommendations (SALSA MLPA Kit P223 PHEX, Versión.01. MRC-Holland, Amsterdam, Netherlands). DNA sequence abnormalities were analyzed in the parents and other relatives of the proband when available.

All the mutations identified in this study were entered into the *PHEX *mutation database [18] (http://www.phexdb.mcgill.ca). A novel missense variation was considered a real mutation and not a genetic polymorphism when it was not found in 130 control chromosomes (65 control females).

### Splicing prediction

Splicing prediction was performed with a module (Alamut Interactive Biosoftware, Rouen, France) which is based in four different algorithms: SpliceSiteFinder, MaxEntScan, NNSplice and GVGD

### Genotype-phenotype correlation

The data considered for the genotype-phenotype correlation in the probands were: gender, severity of skeletal deformities, height SDS, TRP and serum levels of P, 25(OH)D, 1,25(OH)_2_D, PTH and AP before treatment. Presence of nephrocalcinosis and/or hyperparathyroidism before and after treatment was also recorded even though treatment regimens varied across the clinical sites according to local practices. Unfortunately, no phenotypic data were available for some patients.

The mutations detected in the patients were classified into two groups: One group of certain deleterious mutations that resulted in premature stop codons, which included nonsense mutations, insertion or deletion and splice site mutations, and a second group of plausible causative mutations, which included missense mutations and an in frame three nucleotide deletion.

Statistical analysis was performed with the R-free software environment for statistical computing and graphics. The non-parametric Mann-Whitney test was used to compare biochemical levels and height SDS with the specific type of *PHEX *mutation. The Fisher's exact test was used to compare the presence of nephrocalcinosis, hyperparathyroidism and severity of skeletal involvement with the specific type of *PHEX *mutation.

## Results

The underlying *PHEX *mutation was identified in all the 36 probands studied and in their affected family members. One two month old infant belonging to an affected pedigree was also genetically diagnosed prior to clinical diagnosis. Thirty cases (83.3%) appeared to be sporadic because there was no family history of the disease; this group included a pair of monozygotic twins. Only the parents of 21 probands were available for analysis and they were not mutation carriers, which suggested that the mutations had occurred *de novo*. Nevertheless, parental mosaicism could not be totally excluded. Six of the probands were familial cases (16.7%) with at least the mother and one child being clinically affected. In these cases, the identified mutation cosegregated with the disease and was not found in the non affected family members.

### PHEX mutations

**Figure 1:**
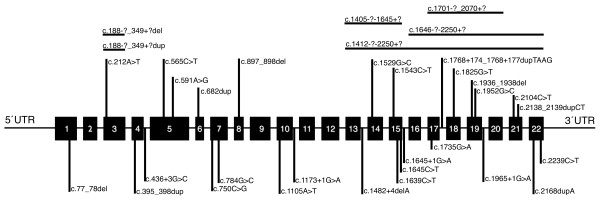
**Schematic representation of the *PHEX *gene with the localization of the mutations found in this study**. Horizontal lines represent the extention of gross deletions. GenBank ID: NM_000444.4

In this study, thirty four different *PHEX *gene mutations were identified. Only two mutations were recurrent in the studied families: the nonsense mutations p.Arg549X, which appeared in two independent cases, and p.Arg702X, which appeared both in one sporadic and in one familial case. All the other mutations were family specific (Table 2). The majority of the mutations found were novel (69.4%) while only nine had been previously described (30.5%) [13,23-26]. Most of the mutations (83.3%) resulted in premature termination codons and therefore in truncated PHEX proteins. These included: nine different nonsense mutations, four of which had been previously described and five were novel. Seven deletions, a pair of two nucleotides deletions and five gross deletions of one single exon or of a group of contiguous exons, which were also novel. One insertion of a single nucleotide, four duplications of one to four nucleotides and one duplication from an unknown site somewhere in intron 2 to somewhere in intron 3 were also found. This last mutation was not detected by genomic DNA sequencing and only became evident by sequencing the RT-PCR product, where exon 3 appeared duplicated, and was thereafter confirmed by MPLA analysis. Seven additional mutations were found to affect the normal splicing and resulted in premature stop codons; six of them were novel. The mutation c.1965+1G > A had been previously described though its effect on the splicing had not been biologically confirmed. In this study, the presence of a pseudoexon which consisted in 59 bp corresponding to intron 19 in the mRNA was proven. Another mutation, c.1654G > A, resulted in a pseudoexon of 74 bp from intron 15. No mRNA of sufficient quality was available from other splicing mutations carriers and the effect of the mutations could not be biologically proven. In those cases, the effect of the mutations was predicted by bioinformatical algorithms (see Materials and Methods). The c.591A > G variant in exon 5 is a silent mutation which encodes for the residue 197 glutamine: however, the theoretical splicing prediction indicated an aberrant splice donor site. This mutation appeared *de novo *in the affected patient. A small number of missense mutations were found, (13.8%), three of them were already described and two were novel, which were confirmed as *de novo *mutations. Finally, there was one in frame deletion, c.1936_1938del that resulted in an mRNA lacking codon 646. All the mutations identified in this series were distributed along the *PHEX *gene although there was a higher mutation density from exon 13 to 22 (Figure 1).

**Table 2:** *PHEX *mutations found in this study

Pr	Inh	E/I	cDNA*	Protein**	Mutation Type	*PHEX*db ID
1	F	E 01	c.77_78del	p.Phe26CysfsX24	Deletion	**261**
2	S	E 03	c.188-?_349+?del	p.?	Del (> 0.16 Kb)	**264**
3	F	E 03	c.188-?_349+?dup	p.Ala63_Lys116dup	Dup (E3)	**262**
4	F	E 03	c.212A > T	p.Asn71Ile	Missense	**263**
5	S	E 04	c.395_398dup	p.Gln133HisfsX14	Duplication	**265**
6	S	I 04	c.436+3G > C	p.?	Splicing	**266**
7	S	E 05	c.565C > T	p.Gln189X	Nonsense	171 [24]
8	S	E 05	c.591A > G	p. =	Splicing	**267**
9	F	E 06	c.682dup	p.Ser228PhefsX10	Duplication	**268**
10	F	E 07	c.750C > G	p.Tyr250X	Nonsense	**269**
11	S	E 07	c.784G > C	p.Ala262Pro	Missense	**270**
12	S	E 08	c.897_898del	p.Lys299AsnfsX5	Deletion	**271**
13	S	E 10	c.1105A > T	p.Arg369X	Nonsense	**272**
14	S	I 10	c.1173+1G > A	p.?	Splicing	**273**
15	S	E 13-15	c.1405-?-1645+?	p?	Del (> 22 Kb)	**274**
16	S	E 13-22	c.1412-?-2250+?	p.Ile495TrpfsX20	Del (> 80 Kb)	**275**
17	S	I 13	c.1482+4delA	p.?	Splicing	**276**
18	S	E 14	c.1529G > C	p.Arg510Pro	Missense	133 [25]
19	S	E 14	c.1543C > T	p.Gln515X	Nonsense	**277**
20	S	E 15	c.1639C > T	p.Gln547X	Nonsense	**278**
21	S	E 15	c.1645C > T	p.Arg549X	Nonsense	13 [23]
22	S	E 15	c.1645C > T	p.Arg549X	Nonsense	13 [23]
23	S	I 15	c.1645+1G > A	p.Phe550fsX21	Splicing	155 [26]
24	S	E 16-22	c.1646-?-2250+?	p.?	Del (> 34 Kb)	**279**
25	S	E 17-20	c.1701-?_2070+?	p.?	Del (> 29 Kb)	**280**
26	S	E 17	c.1735G > A	p.Gly579Arg	Missense	90 [23]
27	S	I 17	c.1768+174_1768+177dupTAAG	p.?	Splicing	**281**
28	S	E 18	c.1825G > T	p.Glu609X	Nonsense	**282**
29	S	E 19	c.1936_1938del	p.Asp646del	Del (in frame)	**283**
30	S	E 19	c.1952G > C	p.Arg651Pro	Missense	76 [13]
31	S	I 19	c.1965+1G > A	p.?	Splicing	62 [23]
32	S	E 21	c.2104C > T	p.Arg702X	Nonsense	16 [23]
33	F	E 21	c.2104C > T	p.Arg702X	Nonsense	16 [23]
34	S	E 21	c.2138_2139dupCT	p.Gln714LeufsX27	Duplication	**284**
35	S	E 22	c.2168dupA	p.Asn723LysfsX3	Duplication	**285**
36	S	E 22	c.2239C > T	p.Arg747X	Nonsense	17 [13]

Pr proband, Inh inheritance, (F familial, S sporadic), E/I Exon/Intron, *[GenBank ID: NM_000444.4], **[Swiss-Prot: P78562], *PHEX*db ID identification in the PHEX mutation database [18] in bold novel mutations

### Genotype-phenotype correlation

The study of any possible correlation between the patient's phenotype and the type of mutation showed that TRP values were significantly lower in patients with clearly deleterious mutations than in patients with plausible causative mutations (61.39 ± 19.76 vs. 80.14 ± 8.80%, p = 0.028). Likewise, 1,25(OH)_2_D serum levels were significantly lower in patients with deleterious mutations in comparison with the other group of patients (40.93 ± 30.73 vs. 78.46 ± 36.27 pg/ml, p = 0.013). On the other hand, no significant correlations were observed between the mutation type and height SDS, serum phosphate, 25(OH)D, PTH or alkaline phosphatase levels at diagnosis (Table 3). Six patients (16%) from this cohort developed nephrocalcinosis and all of them were carriers of deleterious mutations. Unfortunately, the non existence of a standard treatment and the small number of patients with this complication prevents the evaluation of any correlation between treatment regimen and appearance of nephrocalcinosis.

**Table 3:** Genotype-phenotype correlation at diagnosis

Type of mutation	AD	H	P (mg/dl)	TRP (%)	1.25(OH)_2_D (pg/ml)	25(OH)D (ng/ml)	PTH (pg/ml)	ALP (UI/l)
Deleterious (n = 30)	41.18 ± 33.27 (n = 28)	-2.81 ± 1.23 (n = 24)	2.61 ± 0.45 (n = 24)	61.39 ± 19.76 (n = 22)	40.93 ± 30.73 (n = 16)	41.79 ± 42.05 (n = 14)	45.35 ± 17.61 (n = 20)	943.00 ± 451.18 (n = 22)
Plausible (n = 6)	21.83 ± 18.53 (n = 6)	-1.48 ± 1.80 (n = 6)	2.81 ± 0.67 (n = 6)	80.14 ± 8.80 (n = 5)	78.46 ± 36.27 (n = 6)	43.15 ± 19.13 (n = 6)	59.83 ± 17.76 (n = 6)	1114.16 ± 495.77 (n = 6)
*P *values	0.08	0.11	0.53	**0.028**	**0.013**	0.30	0.06	0.48

AD age at diagnosis (months), H height SDS, P phosphate serum levels, TRP tubular reabsorption of phosphate, 1,25(OH)_2_D 1,25 dihydroxi vitamin D, 25(OH)D 25 hydroxy vitamin D, PTH parathyroid hormone, ALP alkaline phosphatase. All parameters were assesed at diagnosis

Eleven patients presented hyperparathyroidism (three before treatment) and ten of them were deleterious mutation carriers. Nevertheless, no significant correlation was observed between the mutation type and the PTH levels at diagnosis. No significant correlation was observed either between the mutation type and the bone phenotype. It should be taken into account that some patients were diagnosed at later ages, which could have worsened their bone phenotype. Finally, phenotype comparison between males and females independent of the mutation type, showed that females developed less nephrocalcinosis than males (p = 0.03). No other significant differences were found between genders.

## Discussion

In this report, the molecular genetic analysis performed in 46 individuals of Iberian ancestry and clinically diagnosed with HR as well as the genotype-phenotype correlation in the probands is presented. The causative *PHEX *mutation was identified both in all the affected individuals from the familial cases (15) as well as in all the sporadic ones (31), thus, confirming a XLHR in the entire cohort. The results obtained confirmed that *PHEX *is the most responsible gene for the HR phenotype in the majority of patients, and that mutations in genes such as *FGF23*, *DMP1 or ENPP1 *must be very scarce in our population. In fact, searching for mutations in other genes was not necessary for the genetic diagnosis in this series.

This study shows, to our knowledge, the highest mutation detection rate reported so far in HR patients. The percentage of detected mutations in other cohort studies ranged from 45% to 87% [13,20,23,26-29]. The high rate of mutation detection in the present study can be explained by several reasons. Firstly, it could be due to the careful clinical and biochemical selection of patients before referring their samples for genetic analysis. Also, the methodology used for the mutation detection could be an explanation. Our approach detects punctual mutations by sequencing but also large deletions or duplications by MLPA analysis. Conversely, researchers of other studies, lack a method for detection of single or contiguous exons deletions. The fact that such type of mutations were not observed in some studies despite a *PHEX *mutation detection rate of 87% in familial and 72% in sporadic cases, underlies the impact of the methodology used. That type of mutations represented 13.9% in our study, with 100% mutation detection rate. Furthermore, it has been pointed out that those single or contiguous exons deletions may account for up to 19% of *PHEX *mutated alleles [30]. Finally, differences in the genetic background between the series published cannot be ruled out. Moreover, in other studies, the mutations detected in familial cases were higher than in sporadic ones, whereas we observed the same mutation rate in both groups.

**Figure 2:**
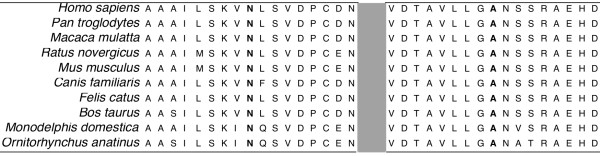
**PHEX protein sequence alignment in different species**. **N **residue asparagine 71 and **A **alanine 262, involved in the p.Asn71Ile and p.Ala262Pro mutations respectively.

All types of mutations were found in this cohort and the majority of them were novel. This was in agreement with other cohort studies where most of the mutations observed were specific to each family and the minority of them was recurrent. Nonsense mutations represented a 30.5% of the causative mutations found in this study. Five were novel and four had been previously described, two of them were recurrent in our study. These mutations resulted in truncated proteins which lack either a large region with the highly conserved metallopeptidase domain or just the final three residues, like the p.Arg747X mutation. The PHEX arginine residue 747 has been shown to be involved in substrate interaction in the homologous protein neprilysin [31]. The nonsense mediated decay, (which selectively eliminates mRNAs containing premature stop codons), is an accepted mediator mechanism in the deleterious process of the nonsense mutations [32]. In fact, we were not able to detect any mRNA corresponding to the nonsense mutated alleles by RT-PCR. Deletions represent a 22.2% of the mutations in this series and all of them were novel: Two small frameshift deletions in exon 1 and 8, and four large deletions which involved several contiguous exons, mainly exons 13 to 22. These mutations were detected by MLPA analysis which was used when no punctual mutation was detected by gene sequencing. In male carriers the deletions were confirmed by the absence of PCR product of the corresponding exons. Two additional in frame deletions were found. A deletion that included the complete exon 3, which would result in a PHEX protein lacking 53 residues of the peptidase M13 and which was considered as deleterious for the genotype-phenotype correlation analysis. A triplet deletion that would result in a protein lacking the aspartic acid 646, which was considered as plausible causative mutation for genotype-phenotype correlation study. Duplications accounted for 13.8% of the mutations. Four were small frameshift duplications and one was a gross duplication that involved the complete exon 3. This last one was evident by RT-PCR and also by MLPA analysis. It is possible that during meiosis a chromatid misalignment occurred and resulted in either deletion or duplication of exon 3 because of more than 85% sequence homology between three stretches of intron 2 and 3. Splicing mutations accounted for 19.4% of the cases. Aberrant mRNA was detected in two of the patients and although no RNA of sufficient quality was available from the rest of them, the effect of the mutation was deduced based on splicing prediction algorithms and cosegregation of the mutation with the disease phenotype. The synonymous mutation c.591A > G created an abnormal splice donor site in the middle of exon 5. Only five missense mutations (13.8%) were found: p.Arg510Pro, p.Gly579Arg and p.Arg651Pro which had been already described associated with the disease [13,20,23,29]. Even more, the p.Gly579Arg mutant protein was shown to be sensitive to endoglycosidase H digestion and to remain trapped in the endoplasmic reticulum in contrast to the wild type PHEX, thus providing a mechanism for loss of PHEX function [33]. And two missense mutations not previously described: p.Asn71Ile and p.Ala262Pro that are very likely pathogenic. Both had appeared *de novo *in the affected individuals and they were not found in 130 normal chromosomes. These mutations resulted in the substitution of highly conserved PHEX aminoacids in different species (Figure 2), which suggested that both residues are important for protein function and that the mutations are probably damaging.

**Figure 3:**
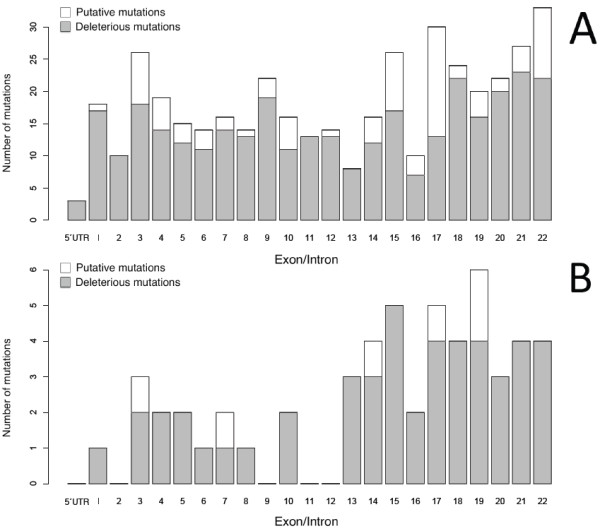
**Distribution of *PHEX *mutations recorded in the *PHEX *locus database **[18]http://www.phexdb.mcgill.ca/**(upper panel) and distribution of mutations found in this study (lower panel)**.

The 36 different mutations found in our study were distributed along the gene and affected almost all exons or their adjacent introns. However, the main density of the mutations was located at the end of the gene, which is one of the three described regions with high mutation density [20]. When comparing the distribution of our mutations with the mutations from the PHEX mutation database [18], a lower mutation density was observed in the first and second part of the gene in our study (Figure 3).

For the genotype-phenotype correlation analysis, the mutations were grouped based on their predicted effect rather than on their localization as it seemed a better approach in other investigations [26,34,35]. One group comprised all the mutations which were very likely deleterious because they would result in a truncated PHEX protein. This group included nonsense mutations, frameshift mutations due to small duplications and deletions, large duplications and deletions and splicing mutations. The second group included missense mutations and a triplet deletion which might result in a PHEX protein with reduced but not totally abolished activity. Indeed, significant correlation between TRP and 1,25(OH)_2_D l serum levels with the mutation type was found. As could be expected, patients with deleterious mutations had lower TRP and lower 1,25(OH)_2_D levels. Both might be the consequence of more active FGF23. Even so, we are conscious that the sample is small, that some parameters may have influenced the results and that larger series of patients should be analyzed to confirm our results. No additional statistical genotype-phenotype correlation was observed with other parameters. The mildest phenotype was shown by the missense p.Ala262Pro mutation carrier. Regarding the bone phenotype, the probands carrying deleterious mutations showed a more severe bone phenotype of no statistical significance, which was in agreement with other studies that reported a trend between truncating mutations and more severe skeletal phenotype [23,34,35]. However, no conclusion can be obtained because patients from the same family and with the same genotype showed bone phenotype differences (data not shown) and on the other hand, it is a subjective assessment made by different physicians and not a quantitative variable. We are aware that great caution should be exercised when assessing genotype-phenotype correlation. In fact, phenotype variation between individuals with the same genotype, particularly regarding the skeletal phenotype, is described [26,36].

To our knowledge, this is the largest study where a correlation between biochemical phenotype and genotype was analyzed. There is a previous study with 8 patients about the genotype correlation with P, AP and TRP which gave no positive results probably due to the small sample size [35].

Regarding the intra-genotype variability, patients 21 and 22 both carry the c.1645C > T (p.Arg549X) mutation and presented very similar phenotype except the development of nephrocalcinosis after treatment. Patients 32 and 33 carry the c.2104C > T (p.Arg702X) mutation and also have similar phenotype except for the development of hyper-PTH after treatment in one and the height SDS unexpectedly positive in the other. Regarding the intra-familial variability, the serum P values and TRP were similar among carriers of the same mutation whilst they showed greater skeletal variability (Data not shown). Nevertheless, the number and size of the families and the number of patients with recurrent mutations was too small to get any conclusive data.

Hyperparathyroidism is rather frequently detected in XLHR patients, which was traditionally explained by chronic phosphorus therapy [37]. However, it should be taken into account that disturbance of the PTH secretion circadian rhythm has been reported among XLHR patients, which increase in nocturnal pulses [38], In addition, animal models of XLHR showed hyperparathyroidism in the absence of treatment [39], and abundant levels of *PHEX *mRNA were observed in the parathyroid gland of two XLH patients [40]. In our cohort, three patients presented hyperparathyroidism before initiating medical therapy and eight more after treatment. Ten of these patients had deleterious mutations. All these data suggested that therapy with phosphorous is not the only cause of hyperparathyroidism in XLHR patients and that more severe *PHEX *mutations may have some influence.

A widespread concern is the occurrence of nephrocalcinosis in the medical treatment of XLHR patients, nearly 80% in some reports. Several risk factors have been identified for its pathogenesis: hypercalciuria, hyperoxaluria and hyperphosphaturia [37]. In our opinion, the degree of phosphaturia can be an important risk factor because all patients in our cohort with nephrocalcinosis had deleterious mutations which in turn were those with less TRP and, therefore, greater phosphaturia. Although the sample size was small and the presence of nephrocalcinosis could be the result of different treatment regimen, our data suggested that the *PHEX *mutation type might be useful to detect patients at increased risk of developing nephrocalcinosis.

## Conclusion

In this report, the genetic analysis of a cohort of HR patients as well as a genotype-phenotype correlation study is presented. The putative *PHEX *mutation was found in all the cases thus confirming genetically the clinical diagnosis. Patients with deleterious mutations presented lower TRP as well as lower 1,25(OH)_2_D serum levels. They seem to be more at risk of developing hyperparathyroidism and nephrocalcinosis than patients with likely causative mutations. Further studies with more patients would be needed to confirm these results. The highest density of mutations was observed in the third last part of the gene. The mutational spectrum as well the percentage of novel and *de novo *mutations was similar to previous reports. Our results confirmed XLHR in all cases and allowed an adequate genetic counselling for the families. We also performed the presymptomatic diagnosis in individuals from affected pedigrees making it possible for them to benefit from prompt therapy.

## Competing interests

The authors declare that they have no competing interests.

## Authors' contributions

MM participated in the conception and design of the study, carried out the genetic studies and the statistical analyses, interpretated data and drafted the manuscript. LC, LS and GA made acquisition, analysis and interpretation of data, revised critically and made substantial contribution to the manuscript. JB, PC, MP, MG, IB, JMD, LR, IR, JN, RV, LM, JV, FA, AF, JMG, SG, BR, SJ, CG, AM, JMM, AM, OM, AF carried out substantial acquisition of data. LL conceived and designed the study and wrote the manuscript, analyzed and interpreted the data and coordinated the data collection. All authors revised the manuscript and gave their final approval.

## Pre-publication history

The pre-publication history for this paper can be accessed here:

http://www.biomedcentral.com/1471-2350/12/116/prepub
